# Development and Comparison of Indirect ELISAs for Detecting IgG and IgA Antibodies Against Major Structural Proteins of Porcine Deltacoronavirus With Virus Neutralization as a Benchmark

**DOI:** 10.1155/tbed/3988285

**Published:** 2025-11-24

**Authors:** Dexin Li, Junhua Deng, Yunjing Zhang, Zenglin Wang, Yufang Li, Liying Hao, Kegong Tian, Xiangdong Li

**Affiliations:** ^1^Jiangsu Co-innovation Center for Prevention and Control of Important Animal Infectious Diseases and Zoonoses, College of Veterinary Medicine, Yangzhou University, Yangzhou, China; ^2^Luoyang Putai Biotechnology Co. Ltd., Luoyang, China; ^3^National Research Center for Veterinary Medicine, Luoyang, China; ^4^Joint International Research Laboratory of Agriculture and Agri-Product Safety, The Ministry of Education of China, Yangzhou University, Yangzhou 225009, China

**Keywords:** IgA, IgG, indirect ELISA, neutralizing antibodies, porcine deltacoronavirus

## Abstract

Porcine deltacoronavirus (PDCoV) is an emerging enteric pathogen that causes substantial economic losses in the global swine industry. Although neutralizing antibodies (NAbs) are a key indicator of vaccine efficacy, their diagnostic concordance with IgG/IgA levels measured by indirect enzyme-linked immunosorbent assays (iELISAs) targeting major structural proteins has not been systematically evaluated. In this study, the full-length spike (S), S1 domain, and receptor-binding domains (RBD) from a highly virulent PDCoV strain were expressed in CHO cells. At the same time, the membrane (M) and nucleocapsid (N) proteins were produced in *Escherichia coli* (*E. coli*). Following initial reactivity screening via protein microarray, S, S1, RBD, and N were chosen to establish iELISAs for detecting IgG and IgA antibodies in serum and milk samples. Evaluating iELISAs' specificity revealed cross-reactivity of anti-S IgA and anti-N IgA with porcine epidemic diarrhea virus (PEDV) antibody-positive sera. Analysis of 75 clinical pig serum samples, and 75 colostrum samples demonstrated that IgA-based iELISAs had superior diagnostic concordance with virus neutralization test (VNT) results than IgG-based iELISAs, with the S1-IgA iELISA showing the highest concordance (93.3%). Furthermore, IgA antibody levels correlated more strongly with neutralizing titers (NTs) than IgG. These findings validate the S1-IgA iELISA as a robust, high-throughput serological tool for assessing PDCoV immunity in pigs.

## 1. Introduction

Coronaviruses (CoVs), enveloped RNA viruses under the subfamily *Orthocoronavirinae* of the family *Coronaviridae* (order *Nidovirales*), are categorized into four genera: *Alpha*-, *Beta*-, *Gamma*-, and *Deltacoronavirus* [1]. They are capable of causing respiratory and gastrointestinal diseases of varying severity in humans and animals. To date, six CoVs have been identified in pigs: transmissible gastroenteritis virus (TGEV), porcine respiratory coronavirus (PRCV), porcine epidemic diarrhea virus (PEDV), and swine acute diarrhea syndrome-coronavirus (SADS-CoV) within the genus *Alphacoronavirus*; porcine hemagglutinating encephalomyelitis virus (PHEV), a member of the genus *Betacoronavirus*; and porcine deltacoronavirus (PDCoV) within the genus *Deltacoronavirus* [2]. PDCoV was first detected in Hong Kong, China, in 2012 and has since been reported in numerous countries [3–8]. Clinical symptoms of PDCoV infection manifest as diarrhea, dehydration, vomiting, and death, often indistinguishable from PEDV or TGEV and frequently complicated by coinfections [9, 10]. Experimental inoculation further proved that PDCoV can infect chickens, calves, and mice [11, 12]. Moreover, it has recently been isolated from the plasma of febrile children, underscoring its potential for cross-species transmission [13].

PDCoV particles are ~100 nm in diameter and possess a genome of ~25.4 kilobases (kb) [8]. The genomic organization of PDCoV is typical of CoVs, comprising a 5′-untranslated region (UTR), multiple open reading frames (ORFs) that encode viral proteins, and a 3′-UTR. The ORFs are arranged in the following order: 5′ -ORF1 a/1b-S-E-M-NS6-N-NS7(NS7a) -3′ [14]. The trimeric spike (*S*) glycoprotein, a type I transmembrane protein, mediates viral entry into host cells. Its ectodomain is proteolytically cleaved into the S1 subunit, which anchors the receptor-binding domain (RBD) responsible for receptor engagement, and the S2 subunit, which facilitates membrane fusion [15]. The nucleocapsid (N) protein, highly conserved among CoVs, is a strongly immunogenic and essential structural component of the virion. Following viral invasion of host cells, abundant expression of the N protein triggers a potent immune response, leading to the extensive production of N-specific antibodies [16]. The membrane (M) protein, the most abundant structural component in the viral envelope, is also highly conserved across PDCoV strains and plays an essential role in virion assembly and morphogenesis [17]. Consequently, the S, M, and N proteins (or their genes) are the principal targets for PDCoV virological and serological diagnostics [10].

Several serological techniques are available for PDCoV detection, including indirect immunofluorescence assay (IFA) [18, 19], virus neutralization test (VNT) [10], fluorescent focus neutralization test (FFNT) [10], and fluorescent microsphere immunoassay (FMIA) [19]. Nonetheless, ELISA has become the first choice because of its simplicity, high cost-effectiveness, and easy use. Various ELISA formats have been developed, including indirect enzyme-linked immunosorbent assays (iELISAs) based on the recombinant N [19, 20], S1 [21, 22], or M [23] proteins, as well as blocking ELISAs employing recombinant N [24] or S [25] proteins. Currently, the diagnostic sensitivity of available ELISA methods is primarily established by comparison with IFA [24–26], whereas the evaluation of vaccine efficacy predominantly relies on the assessment of neutralizing antibody (NAb) titers [15, 27]. Several studies have demonstrated that the coordinated action of PDCoV-specific antibodies following immunization leads to elevated NAb titers, which represent a crucial correlate of protective humoral immunity and a key indicator for vaccine effectiveness [15, 27, 28]. However, the diagnostic concordance, including positive, negative, and overall agreement between iELISA results (detecting IgG/IgA) targeting major structural proteins of PDCoV and NAb status determined by cell-based VNTs, has not been systematically evaluated. Thorough validation of these agreement measures is essential to ascertain the diagnostic accuracy of iELISAs.

In this study, we expressed the full-length S, S1, and RBD proteins in CHO cells, alongside the M and N proteins produced in *Escherichia coli* (*E. coli*). These antigens were employed to develop IgG- and IgA-specific iELISAs for detecting antibodies in serum and milk samples. We subsequently evaluated the specificity, antibody kinetics, and diagnostic concordance of these assays with VNTs using clinical sera, colostrum, and serial serum samples from vaccinated pigs. Our findings provide a critical reference for accurately assessing the immune status in PDCoV-infected or vaccinated swine populations.

## 2. Materials and Methods

### 2.1. Viruses, Cells, Antibodies, and Serum Samples

The PDCoV strain GDSG10-2023 was provided by the National Research Center for Veterinary Medicine. ExpiCHO-S cells, expression medium, and transfection reagent were obtained from Thermo Fisher Scientific (Waltham, MA, USA) and cultured at 32°C under 5% CO_2_. Porcine kidney epithelial cells (LLC-PK1) were cultured in Dulbecco's modified Eagle's medium (DMEM; Gibco, Langley, OK, USA) supplemented with 10% heat-inactivated fetal bovine serum (FBS; Biological Industries, Beit HaEmek, Israel) and maintained at 37°C in a humidified 5% CO_2_ incubator.

The PDCoV monoclonal antibody 1G12 was provided by the National Research Center for Veterinary Medicine. The following commercial antibodies were used: a monoclonal anti–His antibody (CWbio, Beijing, China); fluorescein isothiocyanate (FITC)-conjugated goat anti-mouse IgG (H + L), horseradish peroxidase (HRP)-conjugated goat anti-mouse IgG, and HRP-conjugated goat anti-pig IgA (all from Invitrogen, Waltham, MA, USA); and HRP-conjugated goat anti-pig IgG (Abcam, Waltham, MA, USA).

A total of 108 animal experiment serum samples (40 PDCoV-infected and 68 noninfected), 75 clinical pig serum samples, and 75 sow colostrum samples were provided by the National Research Center for Veterinary Medicine. To validate the applicability of the developed iELISAs for testing milk-derived samples, five PDCoV antibody-positive colostrum from immunized sows were kindly provided by Dr. Bin Li (Jiangsu Academy of Agricultural Sciences). These reference samples were thus used exclusively for this method verification and are distinct from the 75 clinical colostrum samples used in the subsequent diagnostic evaluation. All samples had been previously tested for NAbs.

Standard antibody-positive serum samples against porcine circovirus 2 (PCV2), porcine circovirus 3 (PCV3), porcine reproductive and respiratory syndrome virus (PRRSV), pseudorabies virus (PRV), porcine rotavirus (PoRV), classical swine fever virus (CSFV), and PEDV were provided by Beijing Sino-science Gene Co., Ltd. African swine fever virus (ASFV) antibody-positive serum was purchased from the China Institute of Veterinary Drug Control. Commercial ELISA kits for the detection of PEDV-specific IgG and IgA antibodies, as well as RT-qPCR diagnostic kits for PDCoV and PEDV, were all provided by Luoyang Putai Biotechnology Co., Ltd.

### 2.2. Plasmid Construction

Guided by the recently reported ectodomain topology of the PDCoV S protein [15, 29], the coding sequences of the S, S1, and RBD domains were designed with an added signal peptide to ensure secretory expression, subsequently optimized, synthesized, and cloned into the pcDNA3.1(+) vector. The M-protein sequence was designed by the recently reported structure [17]. The M and N genes were codon-optimized for *E. coli*, synthesized and inserted into pCold-TF and pCold I vectors, respectively (GeneScript, Nanjing, China).

### 2.3. Expression in ExpiCHO-S Cells and Purification

ExpiCHO-S cells were transfected with the recombinant plasmids pcDNA3.1-S, pcDNA3.1-S1, and pcDNA3.1-RBD, respectively. Following transfection, the cells were cultured for 10–12 days. The culture supernatant was harvested and clarified by centrifugation at 5000 × *g* for 30 min at 4°C, followed by filtration through a 0.22-*μ* m sterile membrane. The filtrate was affinity-purified using Ni-NTA agarose resin (GenScript USA Inc., Piscataway, NJ, USA) for 2 h at 4°C. After washing weakly bound impurities with 1 × phosphate-buffered saline (PBS, Solarbio, Beijing, China), the target protein was eluted with 1 × PBS containing 500 mM imidazole. Fractions containing the protein of interest were pooled, and its concentration was determined with a BCA assay kit (Invitrogen, Waltham, MA, USA). Finally, highly pure protein was aliquoted and stored at −80°C for future use.

### 2.4. Expression in *E. coli* and Purification

The recombinant plasmids pCold-TF-M and pCold-I-N were transformed into BL21(DE3) competent cells (TransGen Biotech, Beijing, China), respectively. Protein expression was induced with 0.2 mM isopropyl-β-D-thiogalactopyranoside (IPTG) at 16°C for 12 h. The cells were harvested by centrifugation and lysed by ultrasonication. The lysate was centrifuged, and the supernatant was collected. SDS-PAGE analyzed protein expression. The His-tagged recombinant protein was then purified from the supernatant using Ni-NTA agarose resin according to the manufacturer's instructions. SDS-PAGE was used to analyze the purified protein, and its concentration was determined using a BCA protein assay kit. Finally, the protein was aliquoted and stored at −80°C for future use.

### 2.5. SDS-PAGE and Western Blot

The soluble supernatant and inclusion body fractions from the bacterial lysate, along with the purified proteins, were separated by SDS-PAGE and transferred to a polyvinylidene fluoride (PVDF) membrane (Millipore, Darmstadt, Germany). The membrane was blocked with 5% bovine serum albumin (BSA; Solarbio, China) for 2 h and then incubated with an anti-His monoclonal antibody (diluted 1:2000 in blocking buffer) for 2 h at room temperature (RT). After five washes with PBST (PBS containing 0.05% Tween-20), the membrane was incubated with an HRP-conjugated goat anti-mouse IgG (H + L) secondary antibody (diluted 1:10,000) for 1 h at RT. Following thorough washing with PBST, the proteins were visualized using the Clarity Western ECL substrate (NCM Biotech Co. Ltd., Suzhou, China) and detected with a Western blot imaging system (VILBER Fusion FX7; Vilber Lourmat, Paris, France).

### 2.6. VNT

A fixed virus-diluted serum assay evaluated the NAb titer against PDCoV in LLC-PK1 cells (70%–80% confluence). Serum and milk samples were heat-inactivated at 56°C for 30 min and 60°C for 45 min, respectively. Each sample was serially diluted two-fold (from 1:2 to 1:256) in DMEM containing 10 *μ*g/mL trypsin (Invitrogen, Waltham, MA, USA), with four replicates per dilution. An equal volume of viral suspension containing 200 TCID_50_ was added to each dilution. After gentle mixing, the mixtures were incubated at 37°C under 5% CO_2_ for 1 h. Virus-infected cells (positive control) and negative control cells were included in each assay. Following incubation, 100 *μ* L of each virus-sample mixture was inoculated into the cells in a 96-well plate and incubated at 37°C with 5% CO_2_ for 72 h. The medium was aspirated, and the cells were washed three times with PBS, then fixed with 4% paraformaldehyde (Solarbio, Beijing, China) for 15 min at RT. IFA assessed viral neutralization, and the NAb titer was the highest dilution inhibiting viral infection.

### 2.7. Indirect IFA

The cells were fixed with 4% paraformaldehyde (Solarbio, Beijing, China) for 15 min at RT, then permeabilized with 0.5% Triton X-100 (Solarbio, Beijing, China) for 10 min. After blocking with 1% BSA in PBS for 1 h at RT, the cells were incubated with the primary antibody (PDCoV mAb 1G12; 1:1000 dilution) for 1 h at 37°C. Following three washes with PBS, the cells were incubated with an FITC-conjugated goat anti-mouse IgG (H + L) secondary antibody (1:1000 dilution) for 1 h at 37°C. Fluorescence images were acquired using an inverted fluorescence microscope.

### 2.8. Protein Microarray

The nanomembrane-based protein microarray was constructed and operated using the previously described scoring method [30]. Briefly, proteins were spotted onto activated microarray membranes, with each protein printed at three concentrations (0.3, 0.15, and 0.075 mg/mL). Each subarray included pig IgG (Luoyang Putai Biotechnology Co., Ltd., Luoyang, China) as a quality control. The array was then blocked with 1% BSA for 2 h at RT. After removal of the blocking solution, the microarray was air-dried at 20–25°C for 16–20 h. Finally, the dried microarray was vacuum-sealed and stored at 2–8°C.

A checkerboard titration assay optimized reaction conditions to determine the optimal spotting concentration, serum dilution, and secondary antibody dilution. Serum samples were diluted at 1:25, 1:50, and 1:100, while the HRP-conjugated goat anti-pig secondary antibody was diluted at 1:5000, 1:10,000, 1:20,000, 1:40,000, and 1:80,000. The serum (primary incubation) and secondary antibody were incubated with shaking at 37°C and 500 rpm for 30 min. After washing with PBST, 3,3′, 5,5′-tetramethylbenzidine (TMB) substrate was added and incubated under the same shaking conditions (37°C, 500 rpm) for 15 min. Signal acquisition was performed using a microarray grayscale scanner. The optimal spotting concentration, serum dilution, and secondary antibody dilution for each protein were selected based on the positive-to-negative (P/N) value, and reasonable positive and negative values were chosen as the best reaction conditions of this method. Finally, the optimized conditions were validated for protein reactivity using five PDCoV antibody-positive serum samples and three PDCoV antibody-negative serum samples, with the entire experiment independently repeated three times.

### 2.9. Development of iELISAs

Eight independent iELISAs were developed for the specific detection of IgG or IgA antibodies targeting four recombinant PDCoV proteins (S, S1, RBD, and N). Assay conditions were systematically optimized using a checkerboard titration method with four PDCoV antibody-positive and four negative serum samples, selecting a high P/N ratio and optimal absorbance values.

First, under conventional conditions (30 min incubation for both serum and secondary antibody at 37°C; 15 min TMB development at 37°C), the following parameters were optimized: antigen coating concentration (0.05, 0.1, 0.2, 0.5, 1.0, 1.5, 2.0, 3.0, 4.0, and 5.0 *μ*g/mL), serum dilution ratio (1:25, 1:50, and 1:100), and dilution of HRP-conjugated goat anti-pig IgG/IgA secondary antibody (1:5,000, 1:10,000, 1:20,000, 1:40,000, and 1:80,000). Subsequently, the optimal conditions for antigen coating (2 h at 25°C, 2 h at 37°C, and 24 h at 4°C), blocking conditions (2 h at 25°C, 2 h at 37°C, and 24 h at 4°C), serum incubation time (30, 45, and 60 min at 37°C), secondary antibody incubation time (30, 45, and 60 min at 37°C), and TMB development time (5, 10, and 15 min at 37°C) were determined. Absorbance was measured at 450 nm using a microplate reader (BioTek, VT, USA). All commercial coating buffers, blocking buffers, serum diluent, antibody diluent, and 96-well ELISA plates were supplied by Luoyang Putai Biotechnology Co., Ltd.

### 2.10. Determination of the Cut-Off Values

The cut-off values for all eight iELISAs were established using 108 animal experimental serum samples, including 40 from PDCoV-infected and 68 from noninfected pigs, as detailed in Section 2.1. Receiver operating characteristic (ROC) curve analysis was performed to assess the diagnostic accuracy of the iELISA in differentiating PDCoV-positive and negative serum samples and to establish the optimal cut-off value that maximizes both sensitivity and specificity. Sample-to-positive (S/P) ratios, calculated as (OD_450_ sample − OD_450_ negative control)/(OD_450_ positive control − OD_450_ negative control). For this calculation, the negative control was PDCoV antibody-negative swine sera, and the positive control was an anti-PDCoV serum with an OD_450_ value of ~1.0. The S/P ratios were analyzed using ROC curves generated in GraphPad Prism 8.0 (San Diego, CA, USA). ROC analysis using S/P ratios enabled the calculation of critical diagnostic parameters for each iELISA, namely the optimal cut-off value, sensitivity, and specificity. The optimal cut-off value was selected by maximizing Youden's index based on the sensitivity and specificity derived from ROC analysis [31, 32].

### 2.11. Determination of the Sensitivity, Specificity, and Repeatability

Analytical sensitivity was determined by subjecting a PDCoV antibody-positive control serum to a two-fold serial dilution (from 1:2 to 1:128) and analyzing it under the established iELISA protocol.

Diagnostic sensitivity was assessed using 75 clinical serum and 75 clinical colostrum samples. The NAb status of each sample was first determined via VNT. Subsequently, all samples were tested using the developed iELISA, and the results were compared against the neutralization results.

Specificity was examined using antibody-positive sera against a panel of pathogens, including PCV2, PCV3, PRRSV, PRV, PoRV, CSFV, PEDV, and ASFV. Except for PEDV (20 positive sera), three positive serum samples were tested for each of the remaining pathogens. Additional details regarding these serum samples are provided in Section 2.1.

To evaluate the accuracy of the developed iELISAs for detecting PDCoV antibodies, both intra-assay and inter-assay repeatability were examined. Three antisera showing strong, moderate, and weak positive reactivity, respectively, were analyzed eight times on the same plate to assess intra-assay variation. For inter-assay reproducibility, each antiserum was tested eight times across three independently prepared plates. The coefficient of variation (CV) was calculated as the standard deviation (SD) divided by the mean ($\overset{-}{x}$) for each antiserum to evaluate the precision of the assay.

### 2.12. Animal Experiment

One group of five 4-week-old Large White piglets, confirmed to be negative for PDCoV and PEDV antigens and antibodies by RT-qPCR and ELISA, was used to monitor antibody seroconversion for evaluating the diagnostic sensitivity of the iELISA. All procedures were approved by the Animal Care and Ethics Committee of the National Research Center for Veterinary Medicine (Permit Number 20170012) and were conducted in full compliance with China's Regulations on the Administration of Laboratory Animals (Ministry of Science and Technology) and the ARRIVE guidelines.

An inactivated vaccine was prepared based on a previously described method [15]. Briefly, the PDCoV GDSG10-2023 strain (viral titer: 10^7.3^ TCID_50_/mL) was inactivated with 0.05% β-propiolactone (BPL) at 4°C for 24 h, followed by hydrolysis at 37°C for 2 h. Complete inactivation was confirmed by the absence of cytopathic effect and viral antigen after inoculating LLC-PK1 cells for 4 days, as verified by IFA. The inactivated virus was emulsified with M103 adjuvant (Maximmune, Chengdu, China) to formulate the vaccine. Piglets were intramuscularly immunized with 1 mL of the vaccine. Booster vaccinations were administered following the same protocol at 3 and 6 weeks postprime immunization.

Jugular vein blood was collected from each piglet weekly for serum separation. At week 11 postprime immunization, all animals were euthanized to obtain terminal serum samples.

Euthanasia was performed in accordance with the American Veterinary Medical Association (AVMA) Guidelines. An intravenous overdose of pentobarbital (150 mg/kg body weight; from a 200 mg/mL solution, Merck, Darmstadt, Germany) was administered as a single rapid bolus into the auricular vein using an 18-G catheter. This resulted in immediate unconsciousness (lateral recumbency within 10–15 s), followed by respiratory arrest within 60–90 s and cardiac arrest within 3–5 min. Death was confirmed by the absence of pupillary light reflex and heartbeat on auscultation for a continuous period of at least 5 min. The cessation of all vital signs was independently verified by two licensed veterinarians.

### 2.13. Statistical Analysis

Statistical analyses were performed using GraphPad Prism version 8.0 (San Diego, CA, USA). S/P ratio = (OD_450_ sample − OD_450_ negative control)/(OD_450_ positive control − OD_450_ negative control), where the positive and negative controls were included on each plate. The distribution of the S/P ratio data was assessed for normality using the D'Agostino–Pearson test. Based on this assessment, the Pearson correlation coefficient (*r*) was appropriately selected to evaluate the correlation between iELISA S/P ratios and virus neutralization titers. A *p*-value of <0.05 was considered statistically significant. All graphs were plotted using GraphPad Prism.

## 3. Results

### 3.1. Purification, Identification, and Reactivity Verification of PDCoV Proteins

**Figure 1:**
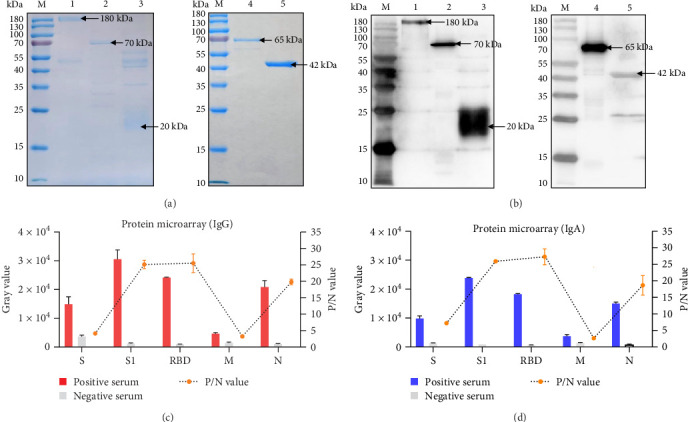
Expression, purification, and reactivity analysis of recombinant PDCoV proteins. (A) SDS-PAGE analysis of purified recombinant PDCoV proteins. Lane M: Protein molecular weight marker; lane 1: S protein (~180 kDa); lane 2: S1 protein (~70 kDa); lane 3: RBD protein (~20 kDa); lane 4: M protein (~65 kDa); lane 5: N protein (~42 kDa). (B) Western blot analysis of the purified proteins using an anti-His tag antibody. Lanes are labeled as in (A). (C–D) Assessment of PDCoV antigen immunoreactivity. Red and blue bars: mean gray values of five PDCoV-positive sera; gray bars: mean values of three negative-control sera; line graph: corresponding P/N ratios.

The recombinant PDCoV S, S1, and RBD proteins were successfully expressed using a eukaryotic CHO cell system, while the prokaryotic system enabled high-yield soluble expression of the recombinant M and N proteins. All recombinant proteins were purified by Ni-NTA affinity chromatography and analyzed by SDS-PAGE and Western blot. SDS-PAGE analysis confirmed the high purity of all proteins, and each protein showed the expected molecular weight (Figure 1A). Western blot analysis using an anti-His tag antibody confirmed the specific immunoreactivity of each purified protein (Figure 1B).

Utilizing protein microarray technology, we evaluated the reactivity of five recombinant PDCoV proteins—S, S1, RBD, M, and N—against specific IgG and IgA antibodies in the samples. As shown in Figure 1C,D, the M protein demonstrated consistently low P/N ratios for detecting M-specific IgG and IgA antibodies, with minimal grayscale variation between positive and negative samples, indicating poor serodiagnosis discrimination. Consequently, the M protein was excluded from further analysis.

S1, RBD, and N exhibited high P/N ratios and clear differentiation between antibody-positive and antibody-negative sera. Although the S protein yielded moderately lower P/N values, it enabled discernible separation between antibody-positive and antibody-negative sera. Based on these reactivity profiles, the recombinant S, S1, RBD, and N proteins were selected for the subsequent assay development.

### 3.2. Establishment and Optimization of iELISAs for PDCoV Antibody Detection

Reaction conditions exhibiting a high P/N ratio and clear discrimination between positive and negative controls were selected as optimal for the iELISA using a checkerboard titration approach. For IgG detection, the optimal coating concentrations for the S, S1, RBD, and N proteins were 0.1, 0.1, 0.1, and 1.5 *μ*g/mL, respectively. Coating and blocking procedures were performed at 4°C for 24 h. Serum samples were uniformly diluted 1:100; the HRP-conjugated goat anti-pig IgG secondary antibody was used at 1:50,000 for the S, S1, and RBD iELISAs and 1:20,000 for the N protein iELISA.

For IgA detection, the optimal coating concentrations for the S, S1, RBD, and N proteins were 2.0, 1.0, 1.5, and 4.0 *μ*g/mL, respectively, with coating and blocking also performed at 4°C for 24 h. Serum was diluted 1:50 for the S, S1, and RBD iELISAs and 1:25 for the N protein iELISA. The HRP-conjugated goat anti-pig IgA secondary antibody was used at 1:10,000 for the S, S1, and RBD iELISAs and at 1:5000 for the N protein iELISA.

For both assays, serum and secondary antibody were incubated for 30 min each at 37°C, with subsequent TMB substrate incubation for 15 min at 37°C in the dark. Colostrum samples were analyzed using the same optimized conditions established for serum.

### 3.3. Determination of Cut-Off Values

Following assay optimization, a panel of 108 porcine serum samples was used to evaluate iELISA performance. Based on VNT results, 40 and 68 samples were classified as PDCoV antibody-positive and -negative, respectively. All samples were tested using the eight developed iELISAs, and the S/P ratios were calculated.

**Figure 2:**
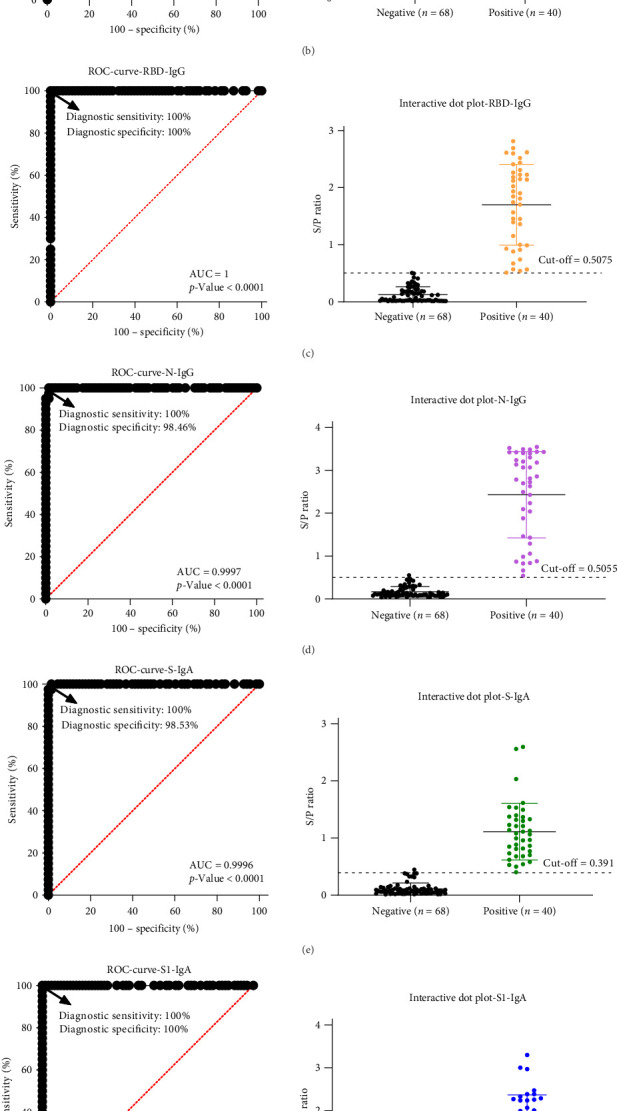
Determination of cut-off values and diagnostic performance of the iELISAs. For each antigen-based assay (A–G), the left panel presents the ROC curve plotting sensitivity against 1-specificity, with the AUC indicating overall accuracy. The right panel displays a dot plot of the S/P ratios obtained from testing known PDCoV-positive and negative serum samples. The horizontal dashed line in each dot plot indicates the optimal cut-off value (determined by maximizing Youden's index).

ROC curve analysis was performed to define optimal cut-off values. For IgG responses (Figure 2A–D), the S-, S1-, and RBD-based iELISAs each achieved an area under the curve (AUC) of 1.000 (*p* < 0.0001; 95% confidence interval (CI): 1.000–1.000), while the N-based IgG iELISA yielded an AUC of 0.999 (95% CI: 0.997–1.000). For IgA responses (Figure 2E–G), the S1 and RBD assays also reached AUCs of 1.000 (95% CI: 1.000–1.000), and the S-based IgA iELISA reached 0.9996 (95% CI: 0.998–1.000).

Sensitivity and specificity were calculated using GraphPad Prism 8.0, with optimal cut-offs selected at the maximum Youden's index. As shown in the interactive dot plots (Figure 2A–G), the cut-offs for IgG-iELISAs were 0.319 (S), 0.3245 (S1), 0.5075 (RBD), and 0.5055 (N); for IgA-iELISAs, they were 0.391 (S), 0.369 (S1), and 0.3455 (RBD). The N protein was excluded from the IgA iELISA ROC analysis due to high nonspecific background reactivity in clinical samples.

### 3.4. Analytical Specificity and Sensitivity

**Figure 3:**
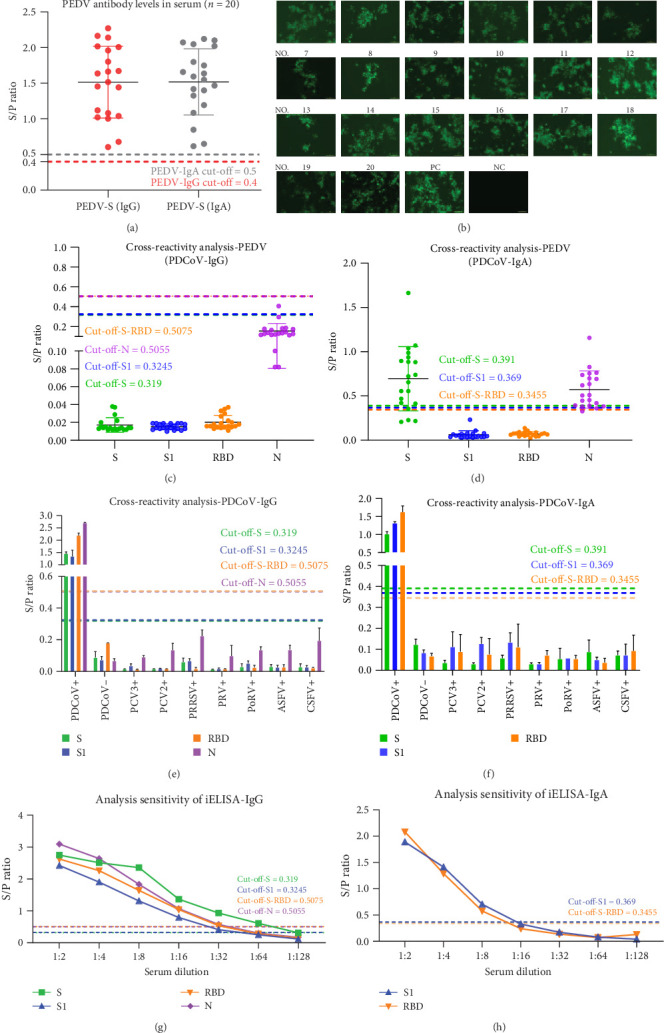
Analytical specificity and sensitivity of the PDCoV iELISAs. (A) PEDV-specific antibody levels in serum, determined by commercial ELISA kits. (B) Neutralizing activity against PDCoV, as determined by VNT. (C, D) Cross-reactivity of the IgG (C) and IgA (D) iELISAs (S-, S1-, RBD-, and N-based) with PEDV-positive serum (*n* = 20). (E, F) Reactivity of the IgG (E) and IgA (F) iELISAs with antisera against other porcine pathogens (PCV2, PCV3, PRRSV, PRV, PoRV, CSFV, ASFV). (G, H) Analytical sensitivity of the IgG (G) and IgA (H) iELISAs, determined by testing two-fold serial dilutions (1:2 to 1:128) of a PDCoV-positive antiserum. The horizontal dashed line in panels (A) and (C–H) indicates the assay-specific cut-off value (determined by Youden's index).

The analytical specificity of the iELISAs was systematically evaluated. A panel of PEDV-positive sera (*n* = 20), which were confirmed for IgG/IgA reactivity by commercial ELISA kits but tested negative for PDCoV-NAbs by VNT (Figure 3A,B), was used. No cross-reactivity was observed with IgG iELISAs (S-, S1-, RBD-, or N-based, Figure 3C).

However, within the IgA group, the S- and N-based iELISAs exhibited cross-reactivity, with the S protein-based assay reacting with 16/20 samples (S/P range: 0.414–1.664) and the N-based assay yielding a maximum S/P ratio of 1.157. In contrast, the S1- and RBD-based IgA iELISAs showed no reactivity (Figure 3D). Furthermore, none of the iELISAs cross-reacted with sera positive for PCV2, PCV3, PRRSV, PRV, PoRV, CSFV, or ASFV (Figure 3E,F). These results indicate that S- and N-based IgA iELISAs exhibit cross-reactivity with PEDV-positive sera, whereas S1- and RBD-based assays demonstrate high analytical specificity.

Analytical sensitivity, determined by two-fold serial dilution of positive sera (Figure 3G,H), was defined as the highest dilution yielding an S/P ratio above the assay-specific cut-off value, revealing that for IgG detection, the S-based iELISA detected antibodies at a dilution of 1:64. At the same time, the S1-, RBD-, and N-based iELISAs were positive up to a dilution of 1:32. For IgA detection, the S1- and RBD-based iELISAs showed positivity only at a dilution of 1:8.

### 3.5. Evaluation of the Repeatability and Reproducibility

The repeatability and reproducibility of each iELISA were assessed by determining intra- and inter-assay coefficients of variation (CV). As shown in Table 1, all iELISAs exhibited intra-assay CVs of 1.2%–6.4% and inter-assay CVs of 3.4%–9.4%, confirming their reliability for PDCoV serodiagnosis.

### 3.6. Diagnostic Sensitivity Against VNT Benchmark

**Figure 4:**
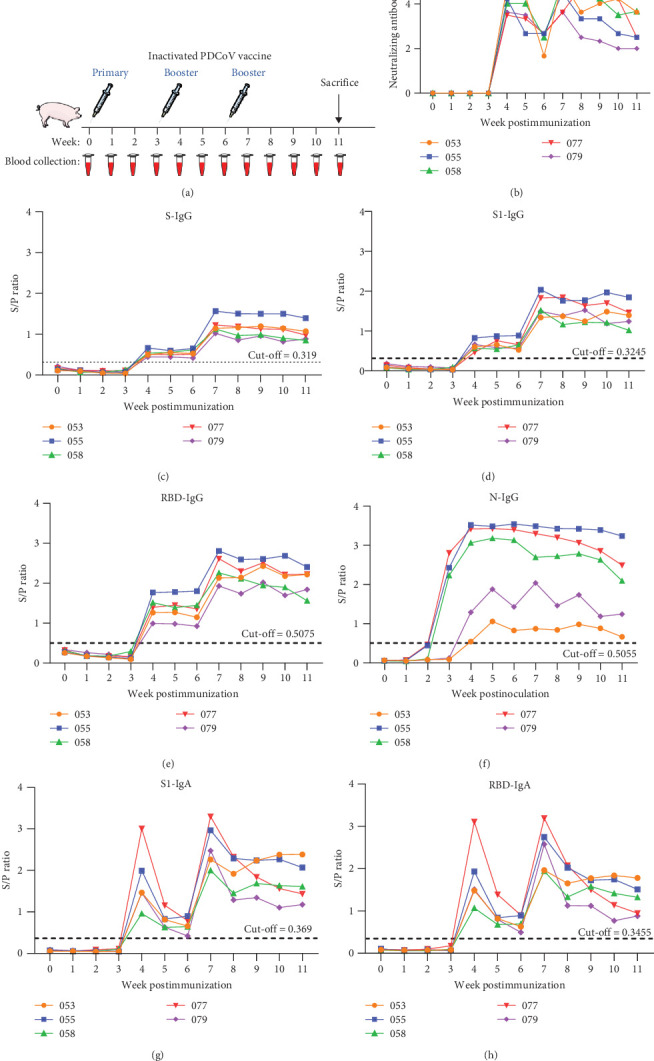
Antibody kinetics in vaccinated pigs and diagnostic sensitivity of iELISAs. (A) Schematic of the animal immunization and sample collection protocol. (B) Neutralizing antibody titers in serum, as determined by VNT. (C–F) Dynamics of IgG antibodies against S, S1, RBD, and N proteins, measured by iELISA. (G,H) Dynamics of IgA antibodies against S1, and RBD proteins, measured by iELISA. The horizontal dashed line in panels (C–H) indicates the assay-specific cut-off value.

To evaluate the diagnostic sensitivity of the established iELISAs, pigs were immunized with an inactivated PDCoV vaccine, and serial serum samples were collected to monitor antibody dynamics (the animal experiment design is outlined in Figure 4A). NAb titers were determined for all collected samples, as shown in Figure 4B.

Analysis of seroconversion kinetics revealed that the N-IgG iELISA detected antibodies as early as 3 weeks postprimary immunization (Figure 4F). In contrast, seroconversion was observed 1-week postbooster for IgG targeting S, S1, and RBD (Figure 4C–E), along with S1- and RBD-specific IgA (Figure 4G,H) and NAbs (Figure 4B).

IgG responses against S, S1, and RBD showed highly concordant kinetics across all five pigs (Figure 4C–E). Similarly, IgA responses to S1 and RBD antigens were uniform. They closely mirrored the biphasic profile of NAbs: an initial rise post-second immunization, a subsequent decline, a second rise post-third immunization, and a final decrease (Figure 4G,H). Despite this consistency within isotypes, IgG and IgA exhibited distinct temporal dynamics.

The diagnostic performance was assessed against a VNT benchmark using 150 clinical samples (Table 2). The S1-IgA iELISA showed the highest total agreement (93.3%), with 98.7% (75/76) positive agreement and 87.8% (65/74) negative agreement. The RBD-IgA iELISA exhibited a total agreement of 90.0%, maintaining the same high positive agreement (98.7%, 75/76) but a lower negative agreement (81.8%, 60/74). Among IgG assays, the S-IgG iELISA showed the highest total agreement (70.7%), with 61.8% (47/76) positive and 79.7% (59/74) negative agreement, indicating limited diagnostic performance. Collectively, IgA-based iELISAs demonstrated superior agreement with VNT compared to IgG-based assays. All individual data are provided in Supporting Information Table S1.

**Figure 5:**
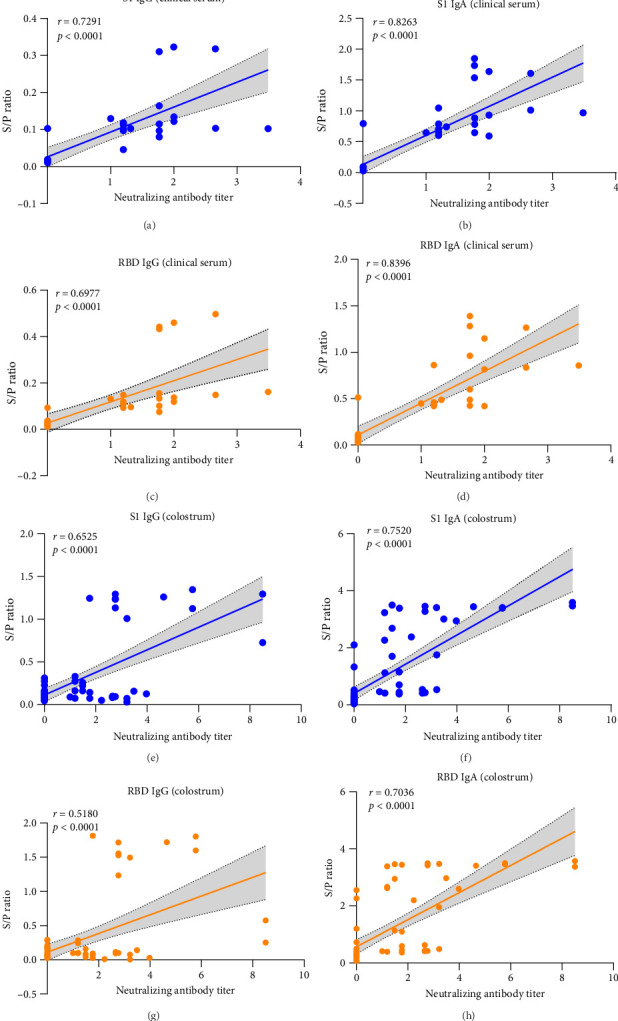
Correlation of iELISA S/P ratios with neutralizing antibody titers. Pearson correlation analysis between S/P ratios (from S1- and RBD-based IgG/IgA iELISAs) and NTs in clinical serum (A–D) and colostrum (E–H) samples. The correlation coefficient (*r*) and statistical significance (*p*-value) are shown for each analysis.

We also evaluated the correlation between S/P ratios (S1- and RBD-based iELISAs) and neutralizing titers (NTs) using 40 clinical serum and 75 colostrum samples. IgA iELISAs correlated more strongly with NTs than their IgG counterparts (Figure 5). All individual data are provided in Supporting Information Table S1.

In summary, integrating results from clinical evaluation (Table 2), antibody kinetics (Figure 4), and correlation analysis (Figure 5), the S1-IgA iELISA demonstrates superior performance and strong potential as a robust diagnostic tool for PDCoV serology.

## 4. Discussion

PDCoV is an emerging coronavirus that threatens the global swine industry and has zoonotic potential. While several ELISA methods for PDCoV antibody detection have been reported, their diagnostic concordance with VNT results—especially across serum, milk, and postvaccination samples—remains unvalidated [19–26]. We systematically developed and evaluated a series of iELISAs targeting major PDCoV structural proteins in the current study, ultimately identifying the S1-IgA iELISA as a highly reliable method that strongly correlates with NAb dynamics and superior agreement with VNT.

Current PDCoV subunit vaccine strategies predominantly focus on the S protein, its S1 subunit, or the RBD [15, 27]. To preserve native conformational epitopes and glycosylation patterns, we expressed the full-length S, S1 subunit, and RBD using a mammalian CHO cell system. Given the enteric tropism of PDCoV, mucosal IgA responses in serum and milk serve as critical indicators of infection or vaccine-induced immunity [26]. We therefore employed high-throughput protein microarray technology to screen five recombinant PDCoV proteins (S, S1, RBD, M, and N) for IgG and IgA reactivity (Figure 1C,D). Considering previous reports of cross-reactivity between PEDV M protein and PDCoV antibodies [33], and given that our own PDCoV M protein showed negligible reactivity and was excluded from subsequent assay development. It is noteworthy that the M protein was produced in a prokaryotic system, and its poor antigenic reactivity in our assay may be influenced by this expression context. While the excellent performance of the *E. coli*-derived N protein demonstrates the general capability of the system; we cannot rule out that a eukaryotic-expressed M protein might exhibit different immunological properties, a possibility that warrants future investigation. This robust screening strategy led to selecting S, S1, RBD, and N proteins for developing IgG- and IgA-specific iELISAs.

Comprehensive evaluation revealed notable limitations in the specificity of specific assays. The S-IgA iELISA exhibited substantial cross-reactivity with PEDV-positive sera (Figure 3D), which was consistent with its poor clinical validation results of 25.7% negative agreement (19/74) with VNT and 55 false-positive readings (Table 2), collectively demonstrating substantial nonspecific reactivity. Similarly, the N-IgA iELISA showed marked nonspecificity, with aberrant signals in VNT-negative samples (precluding cut-off determination) and a maximum OD of 1.157 against PEDV sera (Figure 3D). These findings align with reports of cross-reactive epitopes in the N-terminal region of the nucleocapsid protein [16, 34] and underscore the limited utility of N and full-length S proteins for IgA-specific detection. In contrast, S1- and RBD-based iELISAs exhibited exceptional specificity, supporting their use in targeted serology.

IgA-based assays outperformed their IgG counterparts in sensitivity and clinical correlation. While the full-length S protein provided higher analytical sensitivity in IgG detection (Figure 3G), all IgG iELISAs showed poor positive agreement with VNT (18.4%−61.8%; Table 2). This poor agreement may reflect low IgG concentrations in mucosal samples or insufficient assay sensitivity [26]. Critically, the S1- and RBD-based IgA iELISAs showed strong correlations with NTs in serum samples (S1-IgA: *r* = 0.8263; RBD-IgA: *r* = 0.8386; *p* < 0.001; Figure 5). Furthermore, this correlation was comparable to a previously reported S protein-based cELISA (*r* = 0.861) [25]. This finding reinforces the superior performance of IgA-based assays for PDCoV serology.

Serial sampling of vaccinated animals revealed distinct kinetic profiles across assays. Although N-IgG iELISA detected seroconversion at the earliest post-prime immunization (Figure 4F), its poor clinical concordance with VNT (48%; 62 false negatives) limits diagnostic utility. Importantly, IgA responses against the S1 and RBD antigens closely mirrored the biphasic kinetics of NAbs (characterized by a post-boost rise, subsequent decline, a post-third immunization rise, and a final decline; Figure 4G,H), indicating that IgA dynamics can serve as a valuable surrogate for tracking NAb responses. Moreover, S1- and RBD-IgA iELISAs demonstrated high concordance with VNT (93.3% and 90.0% total agreement, respectively), with 98.7% positive agreement and >81% negative agreement, highlighting their reliability for clinical and vaccine monitoring applications.

## 5. Conclusion

This study conclusively identifies the S1-IgA iELISA as a highly reliable serological tool, demonstrating superior concordance (93.3%) with the VNT and a strong correlation with NAb kinetics. These findings validate the S1-IgA iELISA as a robust, high-throughput alternative for precise evaluation of PDCoV-specific immune responses in both individual pigs and swine populations.

## Figures and Tables

**Table 1:** Repeatability and reproducibility of the established iELISAs.

PDCoV antisera	Intra-assay (IgG/IgA)				Inter-assay (IgG/IgA)			
	S (IgG, %)	S1	RBD	N (IgG,%)	S (IgG, %)	S1	RBD	N (IgG, %)
Strongly positive	2.3	4.5%/1.2%	3.4%/2.5%	1.8	3.4	4.3%/4.2%	5.3%/3.6%	4.0
Moderately positive	3.8	5.3%/3.7%	4.6%/4.3%	4.3	6.5	8.0%/4.8%	6.3%/5.6%	5.2
Weakly positive	4.7	5.0%/4.2%	6.4%/3.8%	4.5	8.6	9.4%/4.8%	6.8%/8.7%	7.6

**Table 2:** Diagnostic performance of iELISAs against the VNT benchmark for detecting PDCoV antibodies in clinical samples (*n* = 150).

Target proteins	a/b/c/d/e/f/g/VNT(+)	a/b/c/d/e/f/g/VNT(−)	a/b/c/d/e/f/g (+)/VNT(+)	a/b/c/d/e/f/g (−)/VNT(−)	a/b/c/d/e/f/g (−)/VNT(+)	a/b/c/d/e/f/g (+)/VNT(−)	Agreement rate (%)
S^a^	62 (41.3%)	88 (58.7%)	47 (61.8%)	59 (79.7%)	29	15	70.7
S1^b^	20 (13.3%)	130 (86.7%)	20 (26.3%)	74 (100%)	56	0	62.7
RBD^c^	17 (11.3%)	133 (88.7%)	17 (22.4%)	74 (100%)	59	0	60.7
N^d^	30 (20.0%)	120 (80.0%)	14 (18.4%)	58 (78.4%)	62	16	48.0
S^e^	130 (86.7%)	20 (13.3%)	75 (98.7%)	19 (25.7%)	1	55	62.7
S1^f^	84 (56.0%)	66 (44.0%)	75 (98.7%)	65 (87.8%)	1	9	93.3
RBD^g^	89 (59.3%)	61 (40.7%)	75 (98.7%)	60 (81.1%)	1	14	90.0
VNT	76 (50.7%)	74 (49.3%)	—	—	—	—	—

*Note:* +, positive; −, negative.

^a^S-IgG iELISA.

^b^S1-IgG iELISA.

^c^RBD-IgG iELISA.

^d^N-IgG iELISA.

^e^S-IgA iELISA.

^f^S1-IgA iELISA.

^g^RBD-IgA iELISA.

## Data Availability

The original contributions presented in the study are included in the article. Further inquiries can be directed to the corresponding authors.

## Supporting Information

Supporting Information Table S1: Summary of S/P ratios and virus neutralization titers for all tested samples.
