# Supplementary material for: Development and Comparison of Indirect ELISAs for Detecting IgG and IgA Antibodies Against Major Structural Proteins of Porcine Deltacoronavirus With Virus Neutralization as a Benchmark
Source: Transbound Emerg Dis. 2025 Nov 24;2025:3988285. doi: 10.1155/tbed/3988285 (PMC12668856; doi:10.1155/tbed/3988285)
Supplement: Supporting Information — Table S1: Summary of S/P ratios and virus neutralization titers for all tested samples. [file 3988285.f1.docx]

**Supplementary data**

**Table S1: Summary of S/P ratios and virus neutralization titers for all tested clinical samples.**

| Samples | NO. | NTs | S/P Ratio | | | | | | |
| --- | --- | --- | --- | --- | --- | --- | --- | --- | --- |
|  |  | (log2) | S-IgG | S-IgA | S1-IgG | S1-IgA | RBD-IgG | RBD-IgA | N-IgG |
| Sera | 1 | 0 | 0.06 | 0.39 | 0.019 | 0.073 | 0.016 | 0.087 | 0.133 |
|  | 2 | 0 | 0.088 | 0.265 | 0.019 | 0.022 | 0.03 | 0.027 | 0.071 |
|  | 3 | 0 | 0.118 | **0.621** | 0.019 | 0.073 | 0.027 | 0.076 | 0.081 |
|  | 4 | 0 | 0.092 | **0.676** | 0.018 | 0.09 | 0.015 | 0.105 | 0.077 |
|  | 5 | 0 | 0.174 | **0.465** | 0.019 | 0.048 | 0.02 | 0.094 | 0.116 |
|  | 6 | 0 | 0.066 | 0.325 | 0.019 | 0.035 | 0.016 | 0.045 | 0.069 |
|  | 7 | 0 | 0.166 | 0.168 | 0.018 | 0.05 | 0.018 | 0.095 | 0.107 |
|  | 8 | 0 | 0.076 | 0.262 | 0.019 | 0.029 | 0.015 | 0.047 | 0.08 |
|  | 9 | 0 | 0.109 | **1.432** | 0.013 | 0.038 | 0.014 | 0.075 | 0.22 |
|  | 10 | 0 | 0.075 | **0.98** | 0.016 | 0.03 | 0.015 | 0.07 | 0.163 |
|  | 11 | 0 | 0.161 | **0.48** | 0.015 | 0.036 | 0.033 | 0.062 | 0.107 |
|  | 12 | 0 | 0.063 | 0.192 | 0.012 | 0.048 | 0.016 | 0.049 | 0.063 |
|  | 13 | 0 | 0.23 | **0.561** | 0.014 | 0.041 | 0.037 | 0.058 | 0.092 |
|  | 14 | 0 | 0.131 | 0.28 | 0.011 | 0.034 | 0.013 | 0.057 | 0.1 |
|  | 15 | 0 | 0.108 | **0.744** | 0.011 | 0.026 | 0.011 | 0.042 | 0.055 |
|  | 16 | 0 | 0.144 | **0.794** | 0.01 | 0.03 | 0.018 | 0.061 | 0.12 |
|  | 17 | 0 | 0.22 | **0.551** | 0.014 | 0.061 | 0.035 | 0.117 | 0.08 |
|  | 18 | 0 | 0.108 | 0.309 | 0.013 | 0.022 | 0.016 | 0.067 | 0.139 |
|  | 19 | 0 | 0.098 | **0.629** | 0.013 | 0.013 | 0.019 | 0.053 | 0.093 |
|  | 20 | 0 | 0.167 | **0.905** | 0.014 | 0.029 | 0.022 | 0.082 | 0.259 |
|  | 21 | 1.77 | **0.596** | **2.924** | 0.309 | **1.733** | 0.433 | **1.282** | 0.097 |
|  | 22 | 2.66 | **0.539** | **2.918** | 0.318 | **1.601** | 0.497 | **1.265** | 0.085 |
|  | 23 | 1.2 | 0.224 | **2.446** | 0.101 | **0.646** | 0.119 | **0.43** | 0.063 |
|  | 24 | 1.2 | 0.204 | **2.639** | 0.096 | **0.782** | 0.11 | **0.459** | 0.105 |
|  | 25 | 1.2 | 0.22 | **2.448** | 0.118 | **1.043** | 0.148 | **0.861** | 0.154 |
|  | 26 | 1.2 | 0.245 | **2.327** | 0.045 | **0.707** | 0.093 | **0.419** | 0.084 |
|  | 27 | 2.66 | 0.099 | **2.362** | 0.103 | **1.009** | 0.148 | **0.835** | 0.155 |
|  | 28 | 1.77 | **0.574** | **2.933** | 0.311 | **1.846** | 0.442 | **1.389** | 0.087 |
|  | 29 | 2 | **0.586** | **3.002** | 0.323 | **1.632** | 0.46 | **1.148** | 0.122 |
|  | 30 | 2 | 0.264 | **2.431** | 0.121 | **0.925** | 0.137 | **0.815** | 0.182 |
|  | 31 | 2 | 0.211 | **2.298** | 0.134 | **0.589** | 0.119 | **0.419** | 0.089 |
|  | 32 | 1.77 | 0.222 | **3.076** | 0.114 | **1.532** | 0.155 | **0.962** | 0.079 |
|  | 33 | 1 | 0.251 | **2.417** | 0.129 | **0.644** | 0.131 | **0.447** | 0.086 |
|  | 34 | 1.77 | 0.218 | **2.797** | 0.08 | **0.775** | 0.075 | **0.424** | 0.127 |
|  | 35 | 0 | 0.202 | **1.922** | 0.102 | **0.79** | 0.093 | **0.512** | 0.122 |
|  | 36 | 1.32 | 0.263 | **2.382** | 0.102 | **0.737** | 0.096 | **0.488** | 0.081 |
|  | 37 | 1.77 | 0.207 | **2.571** | 0.096 | **0.881** | 0.101 | **0.599** | 0.145 |
|  | 38 | 1.2 | 0.288 | **2.31** | 0.113 | **0.597** | 0.114 | **0.439** | 0.085 |
|  | 39 | 1.77 | 0.316 | **2.607** | 0.164 | **0.645** | 0.133 | **0.487** | 0.093 |
|  | 40 | 3.49 | 0.278 | **2.419** | 0.102 | **0.961** | 0.161 | **0.857** | 0.169 |
|  | 41 | + | **0.587** | **1.257** | 0.111 | **0.538** | 0.114 | **0.532** | 0.144 |
|  | 42 | − | 0.228 | 0.318 | 0.066 | 0.192 | 0.157 | 0.309 | 0.132 |
|  | 43 | − | 0.222 | 0.346 | 0.065 | 0.206 | 0.116 | 0.161 | 0.128 |
|  | 44 | − | 0.162 | **0.724** | 0.053 | 0.154 | 0.182 | 0.176 | 0.273 |
|  | 45 | + | **0.406** | **2.483** | 0.202 | **1.221** | 0.202 | **0.662** | 0.135 |
|  | 46 | − | 0.165 | 0.318 | 0.072 | 0.126 | 0.109 | 0.172 | 0.141 |
|  | 47 | + | **0.428** | **2.44** | 0.223 | **1.18** | 0.202 | **0.711** | 0.144 |
|  | 48 | + | **0.493** | **3.055** | 0.236 | **1.749** | 0.275 | **1.051** | 0.132 |
|  | 49 | − | **0.523** | **1.092** | 0.121 | **0.604** | 0.157 | **0.603** | 0.084 |
|  | 50 | − | 0.177 | **1.458** | 0.067 | **0.493** | 0.114 | **0.407** | 0.065 |
|  | 51 | − | 0.094 | 0.134 | 0.058 | 0.119 | 0.142 | 0.094 | 0.084 |
|  | 52 | − | 0.14 | **0.436** | 0.047 | 0.12 | 0.058 | 0.134 | 0.187 |
|  | 53 | − | 0.123 | 0.054 | 0.063 | 0.082 | 0.088 | 0.11 | 0.049 |
|  | 54 | + | 0.156 | **1.853** | 0.124 | **1.109** | 0.211 | **0.854** | 0.082 |
|  | 55 | + | 0.17 | 0.332 | 0.101 | 0.204 | 0.488 | 0.216 | 0.118 |
|  | 56 | + | **0.93** | **3.422** | **0.778** | **3.151** | **3.266** | **2.719** | 0.216 |
|  | 57 | + | **0.349** | **1.793** | 0.231 | **0.855** | 0.178 | **0.583** | 0.091 |
|  | 58 | − | **0.365** | **1.435** | 0.138 | **0.617** | 0.187 | **0.378** | 0.181 |
|  | 59 | + | **0.914** | **3.45** | **0.501** | **3.555** | 0.396 | **3.32** | 0.196 |
|  | 60 | + | **0.38** | **2.38** | 0.145 | **1.348** | 0.168 | **0.688** | 0.13 |
|  | 61 | + | **0.461** | **2.755** | 0.2 | **1.98** | 0.216 | **1.24** | 0.144 |
|  | 62 | + | **0.388** | **3.324** | 0.189 | **2.85** | 0.171 | **2.196** | 0.115 |
|  | 63 | + | **1.305** | **3.347** | **0.867** | **3.382** | **1.234** | **2.989** | 0.404 |
|  | 64 | + | **1.305** | **3.055** | **0.948** | **2.246** | **1.289** | **1.918** | 0.311 |
|  | 65 | + | **1.682** | **3.305** | **1.123** | **3.1** | **1.253** | **2.549** | 0.313 |
|  | 66 | + | **0.662** | **3.378** | 0.28 | **3.165** | 0.264 | **2.746** | 0.247 |
|  | 67 | + | **0.498** | **1.198** | 0.2 | **0.421** | 0.101 | **0.368** | 0.19 |
|  | 68 | + | **0.628** | **1.841** | 0.214 | **0.995** | 0.222 | **0.629** | 0.049 |
|  | 69 | + | **0.326** | **2.507** | 0.234 | **0.76** | 0.1 | **0.591** | 0.16 |
|  | 70 | + | **0.895** | **2.828** | **0.393** | **2.087** | **0.519** | **1.701** | 0.16 |
|  | 71 | + | **0.847** | **3.292** | **0.434** | **3.024** | **0.576** | **2.494** | 0.361 |
|  | 72 | + | **0.483** | **1.775** | 0.187 | **0.777** | 0.162 | **0.517** | 0.148 |
|  | 73 | + | **1.313** | **3.245** | **0.728** | **2.312** | **0.792** | **1.428** | 0.23 |
|  | 74 | + | **0.417** | **3.08** | 0.096 | **1.206** | 0.102 | **0.692** | 0.142 |
|  | 75 | + | **0.651** | **3.277** | 0.322 | **2.731** | 0.307 | **2.075** | 0.12 |
| Colostrum | 1 | 0 | 0.113 | **0.509** | 0.054 | 0.07 | 0.028 | 0.189 | **1.024** |
|  | 2 | 0 | 0.235 | **0.427** | 0.069 | 0.034 | 0.065 | 0.07 | 0.343 |
|  | 3 | 0 | **0.676** | **1.589** | 0.275 | 0.175 | 0.212 | 0.275 | **1.086** |
|  | 4 | 0 | 0.184 | **0.961** | 0.049 | 0.067 | 0.038 | 0.288 | 0.44 |
|  | 5 | 0 | **0.783** | **1.052** | 0.281 | 0.12 | 0.292 | 0.306 | 0.141 |
|  | 6 | 0 | 0.191 | **1.841** | 0.08 | 0.077 | 0.08 | 0.172 | **0.63** |
|  | 7 | 0 | 0.19 | **0.822** | 0.089 | 0.165 | 0.086 | 0.34 | **2.588** |
|  | 8 | 0 | 0.163 | **1.697** | 0.068 | 0.089 | 0.06 | 0.25 | 0.374 |
|  | 9 | 0 | **0.448** | **1.708** | 0.129 | 0.291 | 0.134 | 0.249 | **0.631** |
|  | 10 | 0 | 0.226 | **2.681** | 0.088 | 0.128 | 0.055 | 0.229 | **0.988** |
|  | 11 | 0 | **0.94** | **1.562** | 0.303 | 0.071 | 0.211 | 0.243 | **1.443** |
|  | 12 | 0 | 0.13 | **0.86** | 0.045 | 0.035 | 0.046 | 0.124 | 0.232 |
|  | 13 | 0 | **0.616** | **1.739** | 0.233 | 0.121 | 0.157 | 0.282 | **0.956** |
|  | 14 | 0 | 0.282 | **0.887** | 0.104 | 0.134 | 0.07 | 0.25 | **0.67** |
|  | 15 | 0 | **0.669** | **0.588** | 0.165 | 0.181 | 0.183 | 0.278 | **1.025** |
|  | 16 | 0 | 0.261 | **0.505** | 0.085 | 0.084 | 0.073 | 0.221 | 0.223 |
|  | 17 | 0 | **0.598** | **2.134** | 0.218 | 0.168 | 0.173 | 0.249 | 0.189 |
|  | 18 | 0 | 0.115 | **2.42** | 0.052 | 0.069 | 0.026 | 0.329 | 0.042 |
|  | 19 | 0 | **1.239** | **1.788** | 0.311 | 0.19 | 0.191 | 0.243 | **0.815** |
|  | 20 | 0 | 0.068 | **0.821** | 0.087 | 0.069 | 0.069 | 0.124 | 0.179 |
|  | 21 | 1 | 0.271 | **1.73** | 0.089 | **0.463** | 0.098 | **0.411** | 0.376 |
|  | 22 | 2.77 | **1.53** | **3.645** | **1.247** | **3.443** | **1.234** | **3.439** | **1.868** |
|  | 23 | 1.2 | 0.279 | **1.727** | 0.075 | **0.401** | 0.103 | **0.392** | 0.385 |
|  | 24 | 3.22 | 0.288 | **2.199** | 0.071 | **0.532** | 0.075 | **0.485** | 0.324 |
|  | 25 | 2.77 | **1.582** | **3.615** | **1.131** | **3.424** | **1.528** | **3.422** | **1.762** |
|  | 26 | 2.77 | **1.613** | **3.608** | **1.293** | **3.415** | **1.715** | **3.493** | **1.72** |
|  | 27 | 2.66 | 0.288 | **2.08** | 0.097 | **0.532** | 0.114 | **0.627** | 0.413 |
|  | 28 | 4.65 | **1.509** | **3.576** | **1.257** | **3.429** | **1.72** | **3.406** | **1.789** |
|  | 29 | 2.77 | 0.258 | **1.741** | 0.092 | **0.418** | 0.1 | **0.423** | 0.471 |
|  | 30 | 2.77 | **1.651** | **3.691** | **1.23** | **3.269** | **1.556** | **3.484** | **1.832** |
|  | 31 | 5.78 | **1.536** | **3.697** | **1.12** | **3.407** | **1.599** | **3.498** | **1.717** |
|  | 32 | 2.66 | **0.328** | **1.861** | 0.085 | **0.442** | 0.103 | **0.414** | 0.382 |
|  | 33 | 5.78 | **1.595** | **3.63** | **1.342** | **3.383** | **1.803** | **3.464** | **1.569** |
|  | 34 | 1.77 | **1.657** | **3.62** | **1.242** | **3.376** | **1.813** | **3.44** | **1.002** |
|  | 35 | 0 | 0.264 | **1.762** | 0.092 | **0.428** | 0.107 | **0.431** | 0.387 |
|  | 36 | 1.77 | 0.26 | **1.543** | 0.073 | **0.376** | 0.092 | **0.365** | 0.336 |
|  | 37 | 2.66 | 0.3 | **1.762** | 0.081 | **0.416** | 0.095 | **0.434** | 0.389 |
|  | 38 | 1.77 | 0.241 | **1.87** | 0.075 | **0.442** | 0.073 | **0.445** | 0.367 |
|  | 39 | 3.22 | **1.47** | **3.62** | **1.008** | **3.403** | **1.494** | **3.474** | **1.552** |
|  | 40 | 0 | 0.251 | **1.726** | 0.085 | **0.352** | 0.097 | **0.448** | **0.573** |
|  | 41 | 0 | 0.155 | 0.196 | 0.11 | 0.158 | 0.09 | 0.239 | 0.364 |
|  | 42 | 0 | 0.303 | 0.202 | 0.147 | 0.132 | 0.108 | 0.302 | 0.494 |
|  | 43 | 0 | 0.092 | 0.164 | 0.082 | 0.122 | 0.042 | 0.238 | 0.282 |
|  | 44 | 0 | 0.119 | **1.765** | 0.068 | 0.149 | 0.048 | 0.184 | 0.237 |
|  | 45 | 0 | **0.425** | 0.239 | 0.167 | 0.131 | 0.114 | 0.244 | **0.575** |
|  | 46 | 0 | 0.287 | **2.434** | 0.118 | 0.234 | 0.071 | **0.361** | 0.29 |
|  | 47 | 0 | 0.067 | 0.141 | 0.064 | 0.095 | 0.024 | 0.256 | 0.066 |
|  | 48 | 0 | 0.085 | 0.224 | 0.068 | 0.097 | 0.064 | 0.265 | 0.13 |
|  | 49 | 1.49 | **0.407** | **3.337** | 0.163 | **2.67** | 0.04 | **2.948** | 0.331 |
|  | 50 | 3.49 | 0.238 | **3.392** | 0.155 | **2.991** | 0.141 | **2.973** | 0.121 |
|  | 51 | 1.2 | **0.42** | **3.559** | 0.272 | **3.224** | 0.285 | **3.386** | 0.206 |
|  | 52 | 1.2 | 0.264 | **3.093** | 0.161 | **2.26** | 0.242 | **2.62** | 0.213 |
|  | 53 | 0 | 0.172 | **0.636** | 0.07 | 0.127 | 0.046 | 0.288 | 0.3 |
|  | 54 | 0 | 0.221 | **2.75** | 0.101 | **1.317** | 0.088 | **1.201** | 0.126 |
|  | 55 | 1.49 | **0.43** | **3.426** | 0.221 | **1.69** | 0.159 | **1.133** | **0.535** |
|  | 56 | 0 | **1.464** | **2.929** | 0.102 | 0.199 | 0.061 | 0.336 | 0.373 |
|  | 57 | 0 | 0.065 | **2.708** | 0.064 | 0.105 | 0.034 | 0.277 | 0.09 |
|  | 58 | 0 | **0.495** | **3.314** | 0.237 | **2.092** | 0.282 | **2.554** | 0.152 |
|  | 59 | 0 | 0.091 | **2.353** | 0.075 | 0.069 | 0.032 | **2.264** | 0.066 |
|  | 60 | 0 | 0.293 | **1.901** | 0.14 | 0.129 | 0.109 | 0.23 | 0.254 |
|  | 61 | 0 | 0.222 | **1.297** | 0.083 | 0.096 | 0.048 | 0.247 | 0.263 |
|  | 62 | 0 | 0.294 | **3.045** | 0.122 | 0.148 | 0.059 | **0.352** | 0.252 |
|  | 63 | 0 | 0.136 | **0.518** | 0.091 | 0.212 | 0.054 | **0.502** | **0.527** |
|  | 64 | 1.2 | **0.917** | **3.458** | **0.329** | **1.113** | 0.096 | **2.667** | **0.813** |
|  | 65 | 0 | **0.421** | **2.653** | 0.085 | 0.267 | 0.015 | **0.725** | **0.509** |
|  | 66 | 1.49 | **0.67** | **3.591** | 0.258 | **3.493** | 0.085 | **3.46** | 0.076 |
|  | 67 | 3.22 | 0.059 | **3.234** | 0.031 | **1.74** | 0.006 | **1.951** | 0.032 |
|  | 68 | 0 | **0.453** | **3.338** | 0.096 | **0.519** | 0.015 | **0.369** | 0.423 |
|  | 69 | 1.77 | **0.454** | **2.946** | 0.14 | **0.69** | 0.026 | **0.592** | **0.726** |
|  | 70 | 2.23 | 0.177 | **3.355** | 0.049 | **2.369** | 0.01 | **2.197** | **0.521** |
|  | 71 | 1.77 | **0.882** | **2.782** | 0.075 | **1.149** | 0.018 | **1.098** | 0.251 |
|  | 72 | 8.5 | **1.387** | **3.628** | **0.724** | **3.456** | 0.253 | **3.37** | 0.297 |
|  | 73 | 8.5 | **1.86** | **3.526** | **1.291** | **3.587** | **0.575** | **3.573** | **2.711** |
|  | 74 | 3.98 | **0.362** | **3.538** | 0.124 | **2.931** | 0.029 | **2.599** | 0.029 |
|  | 75 | 0 | 0.267 | **1.567** | 0.103 | 0.067 | 0.019 | 0.174 | **0.58** |

**Note:** Bold values indicate positive results based on established cut-off criteria;

+: positive; −: negative;

**Table Caption:** Statistical data of S/P ratios (determined by established indirect ELISAs for PDCoV-S-IgG, S-IgA, S1-IgG, S1-IgA, RBD-IgG, RBD-IgA, and N-IgG) and neutralizing antibody titers/results obtained from 75 clinical serum and 75 milk samples. This dataset was used for the sensitivity analysis of the ELISA method (Table 2) and the correlation analysis (Figure 5).
